# Low Temperature Synthesis of Phase Pure MoAlB Powder in Molten NaCl

**DOI:** 10.3390/ma13030785

**Published:** 2020-02-09

**Authors:** Cheng Liu, Zhaoping Hou, Quanli Jia, Xueyin Liu, Shaowei Zhang

**Affiliations:** 1 College of Engineering, Mathematics and Physical Sciences, University of Exeter, Exeter EX4 4QF, UK; foxbat25mig@163.com; 2 College of Materials Science and Engineering, Taiyuan University of Technology, Taiyuan 030024, China; houzhaoping@tyut.edu.cn; 3 Henan Key Laboratory of High Temperature Functional Ceramics, Zhengzhou University, Zhengzhou 450052, China; jiaquanli@zzu.edu.cn; 4 College of Civil Engineering and Architecture, Quzhou University, Quzhou 32400, Zhejiang, China

**Keywords:** MoAlB, molten salt synthesis, low temperature, NaCl, boron

## Abstract

MoAlB fine powders were prepared in molten NaCl from Al, B and Mo powders. The effects of key parameters affecting the synthesis process and phase morphology were examined and the underpinning mechanisms proposed. MoAlB product particles exhibited different shapes/sizes, as follows: spherical grains (1~3 μm), plate-like particles (<5 μm in diameter) and columnar crystals with lengths up to 20 μm and diameters up to 5 μm, resultant from different reaction processes. Phase pure MoAlB was synthesised under the following optimal conditions: use of 140% excess Al and 6 h of firing at 1000 °C. This temperature was at least 100 °C lower than required by other methods/techniques previously reported. At the synthesis condition, Mo first reacted with Al and B, forming Al_8_Mo_3_ and MoB, respectively, which further reacted with excess Al to form Al-rich Al–Mo phases and MoAlB. The Al-rich Al–Mo phases further reacted with the residual B, forming additional MoAlB. The molten NaCl played an important role in accelerating the overall synthesis process.

## 1. Introduction

Ternary transition metal borides have layered structures similar to MAX phase materials [1] and are also regarded as a new class of promising non-oxide ceramics. Among them, MoAlB has attracted particular attention because of its many excellent properties and great application potential (e.g., electrocatalysis, composite reinforcement, solid lubrication and high temperature coating). It has a good electrical/thermal conductivity, small thermal expansion, great compressive strength and high fractural toughness [2,3,4,5,6,7]. It also has been confirmed to be stable at up to 1400 °C in an inert atmosphere [3]. Differently from in MoB, the Al atoms in MoAlB are “sandwiched” between the MoB layers, which renders MoAlB very oxidation resistant (via the formation of a dense alumina layer from the initial oxidation on heating) [2,3,8,9]. 

Bulk or powder-formed MoAlB can be synthesised by using various techniques/methodologies, including the Al flux method, spark plasma sintering (SPS), hot pressing and conventional mixed powder technique. The Al flux method [1,10,11,12] requires a very high operation temperature (between 1400 and 1800 °C), and is mainly used to synthesise MoAlB single crystals (>100 μm). SPS [13] and hot pressing [3,8,9] require a lowered firing temperature (1100~1200 °C) but a high pressure. They are mainly used to prepare bulk MoAlB from MoB and Al powders. To our knowledge, the only method that has been attempted to date to synthesise MoAlB powder is the conventional mixed powder technique [6,14], i.e., directly heating a mixture of MoB and Al at a high temperature (>1100 °C). Unfortunately, with this technique, it is difficult to prepare high quality (phase pure, good dispersion and fine size) MoAlB powders, because some intermediate/impurity phases (e.g., MoB_2_) almost always remain in the final product powder. 

In the work reported here, a liquid salt assisted synthesis method was used to prepare MoAlB fine powder from Mo, Al and B powders. The main factors affecting the synthesis process and product’s microstructure/morphology were examined and the underpinning mechanisms discussed.

## 2. Experimental Procedure

### 2.1. Raw Materials and Sample Preparation 

Al (purity 99.7%, overall size < 25 μm), amorphous B (purity 95%), and Mo (purity ≥ 99%, overall size < 250 nm) powders from Sigma-Aldrich were utilized as the starting materials, and NaCl (≥99%) was used to form a liquid medium. The three raw materials were mixed in the stoichiometric ratios corresponding to Reaction 1 (i.e., 0.96 g Mo, 0.108 g B and 0.27 g Al), or non-stoichiometric ratios with excess Al, followed by further combination with 20 g NaCl in an agate mortar. The powder mix was contained in a covered graphite crucible, placed in a tube furnace and then heated in argon at 5 °C/min to a given temperature within 850–1200 °C. After 6 h at the temperature, the sample was furnace-cooled to room temperature.

Mo + Al + B = MoAlB(1)

The residual salt in the fired samples was removed by repeated water-washing. For some samples, a 6M HCl solution was further used to leach out the residual Al, followed by rinsing with distilled water. All of the resultant samples, after oven-drying overnight at 100 °C, were subjected to detailed characterisation.

### 2.2. Sample Characterisation

The phases in the fired/purified samples were identified based on X-ray diffraction (XRD) analysis. The diffractometer (Brukers D8 advance reflection diffractometer, Karlsruhe, Germany) was operated at 40 mA/40 kV, with Ni-filtered CuKα radiation and at the scan rate of 2.4° (2θ)/min with an interval of 0.04°. The following International Centre for Diffraction Data (ICDD) cards were used to identify the corresponding phases: MoB (51-0940), Al_2_O_3_ (46-1212), Al (65-2869), MoAlB (65-2497), Al_5_Mo (44-1102), Al_4_Mo (65-7072) and Al_8_Mo_3_ (65-1231). The microstructures and morphologies of the samples were characterised using a scanning electron microscope (SEM Nova Nanolab 600, FEI Company, Hillsboro, OR)) along with a JEM 2100 transmission electron microscope (TEM).

## 3. Results 

### 3.1. Effect of Firing Temperature on MoAlB Formation

**Figure 1:**
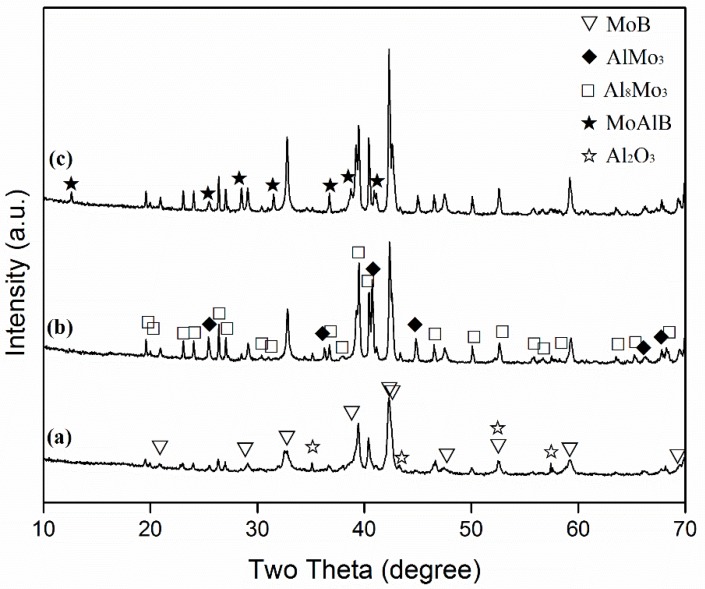
XRD spectra of the samples with the stoichiometric composition after 6 h at (**a**) 850, (**b**) 900 and (**c**) 950 °C.

Figure 1 gives the XRD results of the samples with the stoichiometric composition after 6 h at 850–950 °C. At 850 °C, Al_8_Mo_3_ and MoB were formed as the primary phases, along with minor intermediate AlMo_3_ and impurity Al_2_O_3_ (the latter was likely resultant from the minor oxidation of Al by the impurity of O_2_ in the Ar gas), but no MoAlB was detected (Figure 1a). Upon increasing the temperature to 900 °C, the same phases were detected, and their contents increased evidently, in particular, in the case of AlMo_3_. However, MoAlB was still not detectable by XRD (Figure 1b). Upon further increasing the temperature to 950 °C, AlMo_3_ disappeared and MoAlB started to appear. Mo_8_Al_3_ and MoB were also formed, and the content of the latter became slightly higher than at 900 °C (Figure 1c). As a result of the formation of intermediate Mo–Al phases, most of the Al had been consumed. Consequently, only small amounts of free Al were available for MoAlB formation, which explained the limited formation of MoAlB at this temperature (Figure 1c). 

### 3.2. Effect of Amount of Excess Al on MoAlB Formation

**Figure 2:**
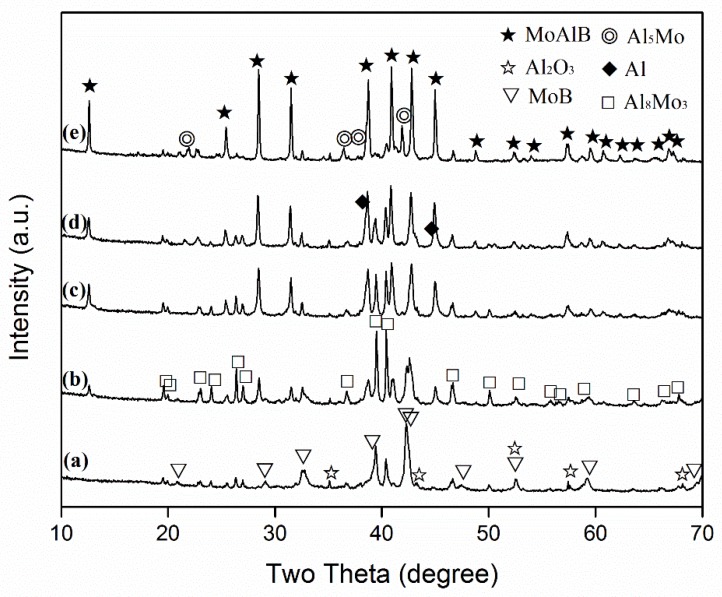
XRD spectra of the samples heated at 850 °C for 6 h, with different amounts of excess Al at (**a**) stoichiometric amount, (**b**) 60%, (**c**) 80%, (**d**) 100% and (**e**) 120%.

Figure 2 illustrates the effect of excess Al on the phase formation and reaction extent in the samples after 6 h of firing at 850 °C. When the stoichiometric amount of Al was used (Figure 2a, i.e., Figure 1a), as already stated above, no MoAlB was detected, and Al_8_Mo_3_ and MoB were formed as the primary phases, along with minor impurity Al_2_O_3_. Upon increasing the excess Al to 60%~80%, the formation of MoAlB became evident, but MoB and Al_8_Mo_3_ still remained as the main phases (Figure 2b,c). Upon further increasing the excess Al to 100–120%, MoAlB further increased, whereas MoB and Al_8_Mo_3_ generally decreased. In addition, minor Al_5_Mo was detected (Figure 2d,e). The above results indicated that increasing the excess Al generally favoured MoAlB formation, but inhibited the formation of intermediate phases. The intermediate phases detected were mainly in the Al–Mo binary phases along with minor MoB, indicating that there should be some unreacted B (although not detectable by XRD because of its amorphous nature) remaining in all of the samples. The formation of Al–Mo phases in all of the samples indicated that they were stable in the presence of B at 850 °C. Therefore, a higher synthesis temperature had to be used to avoid their formation and to complete the MoAlB formation reaction.

### 3.3. Combined Effects of Excess Al and Firing Temperature on MoAlB Formation, and Optimisation of Synthesis Condition

**Figure 3:**
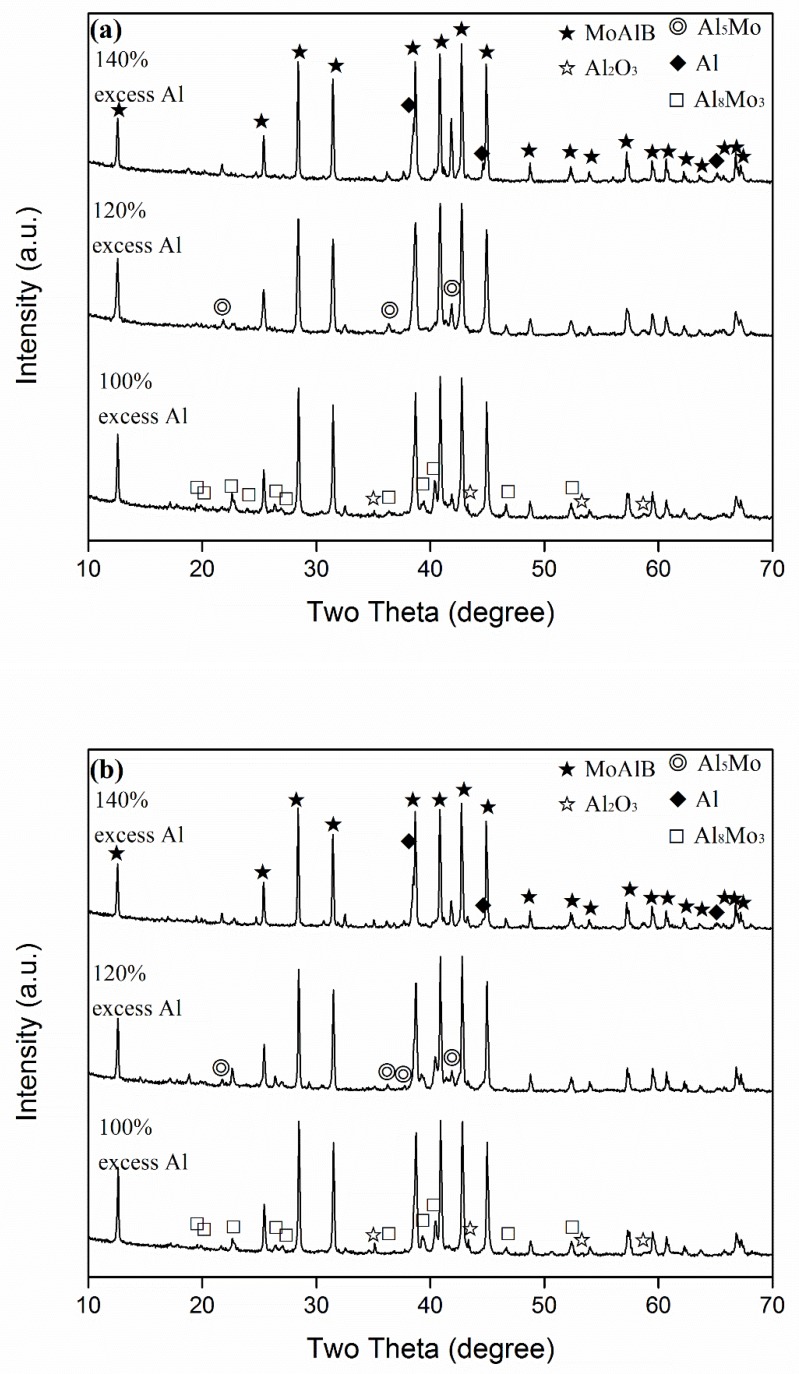
XRD patterns of the samples with various amounts of excess Al, after 6 h of firing at (**a**) 900 and (**b**) 950 °C, separately.

**Figure 4:**
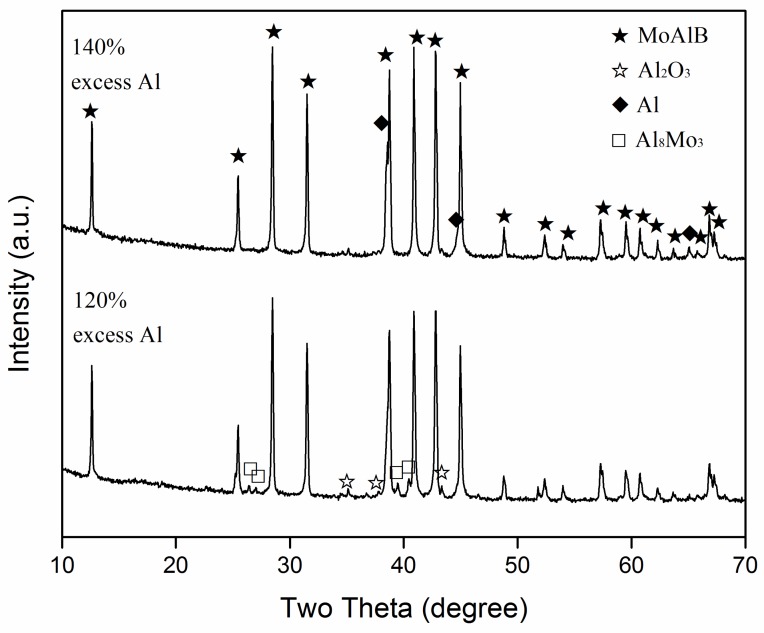
XRD patterns of samples with 120% and 140% excess Al, separately, after 6 h of firing at 1000 °C.

**Figure 5:**
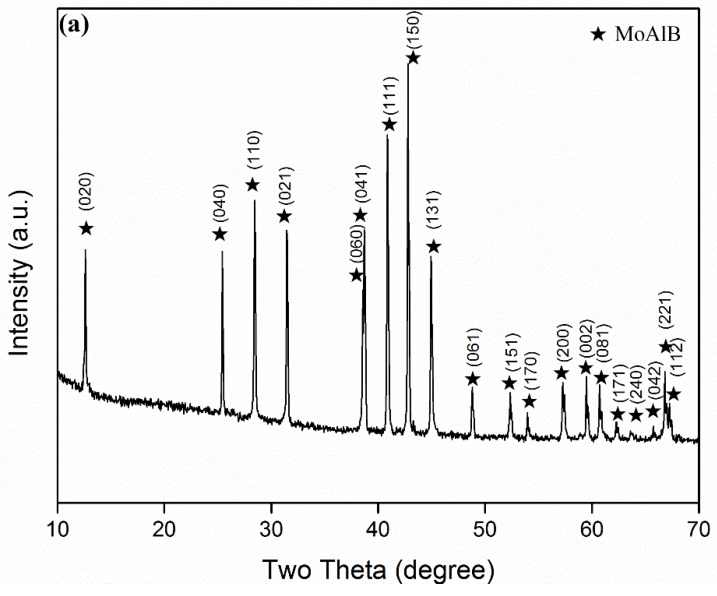

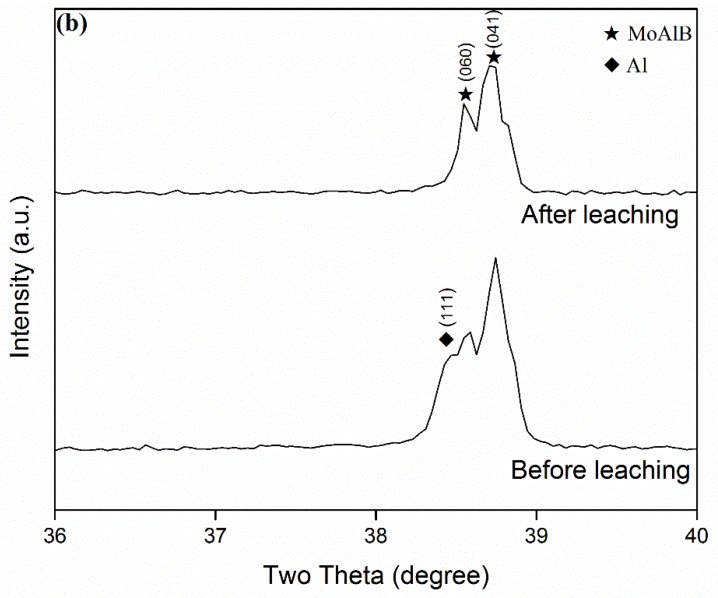
(**a**) XRD pattern of the sample after 6 h of firing at 1000 °C and the subsequent acid leaching, and (**b**) comparison of XRD patterns (within 36–40°) of the samples before and after acid leaching.

To illustrate the combined effects of excess Al and the firing temperature on the phase formation and reaction extent, and to further optimise the synthesis condition, samples with various amounts of excess Al were fired at different temperatures. Figure 3, for example, shows the XRD patterns of samples with 100–140% excess Al after 6 h of firing at 900 and 950 °C, respectively, revealing similar phase formations in both cases, although slightly more MoAlB and less intermediate phases were formed at 950 than at 900 °C. When 100% excess Al was used, Al_8_Mo_3_ and MoB were the main intermediate Mo–Al phases. However, with increasing the excess Al, these two phases decreased, but an Al-rich Al–Mo phase, Al_5_Mo, was detected. The above results revealed that out of all of the samples, the one with 140% excess Al had the highest MoAlB formation and the least formation of intermediate Al–Mo phases at 950 °C, further indicating that increasing the excess Al along with firing temperature had a synergistic effect on MoAlB formation. So, to make even purer MoAlB, samples with 120% and 140% excess Al were further fired at 1000 °C for 6 h. As seen from Figure 4, when 120% excess Al was used, apart from the primary phase of MoAlB, only minor Al_8_Mo_3_ and residual Al were detected. However, upon increasing the excess Al to 140%, all of the intermediate Al–Mo phases disappeared, and nearly phase pure MoAlB, along with minor residual Al and minor Al_2_O_3_, was formed. After leaching out the minor residual Al and Al_2_O_3_ with HCl, phase pure MoAlB was finally obtained (Figure 5a). The elimination of Al was evidenced by the absence of its diffraction peaks, e.g., the strongest one at about 38.5^o^ (2θ) (Figure 5b). The synthesis temperature used here was at least 100 °C lower than that required by other synthesis routes reported previously [1,3,8,9,10,11,12,13]. 

### 3.4. Microstructural Characterisation of MoAlB Product Powder

**Figure 6:**
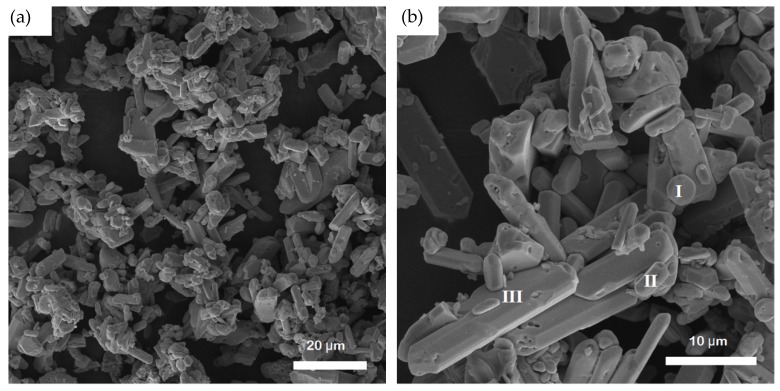
(**a**) Low and (**b**) high magnification SEM images of MoAlB particles resulting from 6 h of firing at 1000 °C and the subsequent acid leaching.

Figure 6 shows the SEM images of the MoAlB product powder resultant from 6 h of firing at 1000 °C and the subsequent purification with HCl, revealing three different morphologies of the particles, namely: small rounded grains of 1~3 μm (highlighted by “I” in Figure 6), platelet-like (disc-like) particles of <5 μm in diameter (highlighted by “II” in Figure 6) and columnar crystals up to 20 μm in length and up to 5 μm in diameter (highlighted by “III” in Figure 6). These three forms of particles looked similar to those prepared by Shi et al. via the conventional mixed powder route using Al and MoB as the raw materials [15]. Some small pits were seen on the surfaces of the third form, which were similar to those reported by Kota and Shi et al. [9,15], although the reason behind their formations was not clear. 

**Figure 7:**
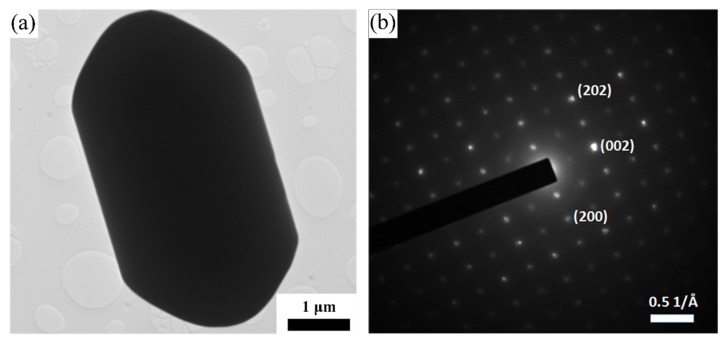
(**a**) TEM image and (**b**) selected area electron diffraction (SAED) of a representative MoAlB crystal in the sample, whose microstructure is shown in Figure 6.

Figure 7a further presents a TEM image of a representative MoAlB particle from the sample whose microstructure is shown in Figure 6. As a result of its micron-scale thickness, its lattice structure could not be revealed under TEM. Nevertheless, the selected area electron diffraction (SAED) pattern of the particle was obtained (Figure 7b). It was similar to that reported by Alameda et al. [16], confirming that the particle was an MoAlB crystal, which is believed to be as a result of the preferential growth in the [010] direction.

## 4. Discussion

At the test temperatures, the NaCl (melting point ~714 °C) melted, forming a homogeneous molten salt medium in which the Al (melting point 660.3 °C) melted, forming corresponding droplets. Although the exact solubility data of the Al, Mo and B in the NaCl molten salt are not available, based on the previous studies on the molten salt synthesis of Mo_2_C [17], borides [18,19,20] and TiB_2_–Al_2_O_3_ [21], it can be considered that the solubility values should be in the following order: Mo> Al> B. At a relatively low firing temperature (850 °C), Mo was partially dissolved in the liquid salt, diffused quickly through it onto the Al droplets (or met with dissolved Al) and B particles and then reacted to form intermediate phases Al_8_Mo_3_ and MoB according to Reactions (2) and (3), respectively (Figure 1a and Figure 2a).

8Al + 3Mo = Al_8_Mo_3_ (ΔG° = −278.7 KJ at 1000 °C) (2)

Mo + B = MoB (ΔG° = −110.4 KJ at 1000 °C) (3)

No intermediate Al–B phases (such as AlB_2_) were detected by XRD at this temperature (Figure 1a and Figure 2a). The reason might be that most of the Al had been consumed by the reaction with Mo, so almost no Al was left for its reaction with B. Because of this and the limited solubility of B in the molten salt at this relatively low temperature, once Al_8_Mo_3_ and MoB were formed, it would be difficult for them to be converted into MoAlB via reacting with B and Al, respectively, i.e., Reactions (4) and (5) would not occur at this low temperature. This explained the absence of MoAlB in the sample with the stoichiometric amount of Al after firing at 850 °C (Figure 1a and Figure 2a).

Al_8_Mo_3_ + 3B = 3MoAlB + 5Al (4)

MoB + Al = MoAlB(5)

With increasing the amount of excess Al, MoAlB increased, whereas MoB decreased, indicating the occurrence of Reaction (5) and its gradually enhanced extent (Figure 2). Apart from this, some of the excess Al would react with the Al_8_Mo_3_ formed earlier, forming Al-rich Al–Mo phases (Al_x_Mo), as suggested by the Al–Mo phase diagram [22] and evidenced by the detection of Al_5_Mo (Figure 3). Al_5_Mo was not stable at the test temperatures, so it was believed to be formed upon cooling from the liquid with the same composition. This was responsible for the increased Al_5_Mo and decreased Al_8_Mo_3_ upon using more excess Al (Figure 2 and Figure 3).

Al_8_Mo_3_ + 7Al = 3Al_5_Mo (ΔG° = −23.5 KJ at 1000 °C) (6)

Al_x_Mo + B = MoAlB + (x − 1)Al (7)

In addition to the amount of excess Al, the firing temperature significantly affected MoAlB formation, in cases both using and without using excess Al (Figure 1, Figure 3 and Figure 4). With increasing the firing temperature, the solubility of B in the molten salt would be increased. So, dissolved B would diffuse through the molten salt onto the remaining Al–Mo phases (Al_8_Mo_3_ and Al_x_Mo), and reacted with them to form MoAlB, according to Reactions (4) and (7) (Figure 1c, Figure 3 and Figure 4). 

Based on the above discussion, it can be considered that phase pure MoAlB could only be prepared by using appropriate amounts of excess Al and firing at an appropriate temperature (in the present work, 140% excess Al at 1000 °C). It is believed that the multiple-step reactions, in particular, the three different formation reactions (Reactions (4), (5) and (7)), were responsible for the different shapes and sizes of the MoAlB product particles (Figure 6 and Figure 7), although future work is still needed to classify this further. 

## 5. Conclusions

MoAlB fine particles were synthesised in molten NaCl at a lowered temperature, from Al, B and Mo powders. The main conclusions are drawn as follows.

(1) MoAlB product particles exhibited three different shapes/sizes, namely: rounded particles (1~3 μm), platelet-like particles (<5 μm in diameter) and columnar crystals with various lengths (up to 20 μm) and diameters (up to 5 μm), which are believed to be formed from different reaction processes.

(2) To prepare phase pure MoAlB, an appropriate amount of excess Al needs to be used and an appropriate firing temperature is required. In the present work, the optimal synthesis conditions were as follows: use of 140% excess Al and 6 h of firing at 1000 °C. This synthesis temperature was at least 100 °C lower than that required by the synthesis techniques reported previously.

(3) At the test temperatures, the NaCl and Al melted, forming a liquid pool and corresponding droplets, respectively. If the temperature was low (at 850 °C), Mo and Al partially dissolved in the liquid salt, but B did not dissolve. In this case, the dissolved Mo reacted with Al (dissolved and/or undissolved) and undissolved B, forming intermediate Al_8_Mo_3_ and MoB, respectively. When excess Al was used, it was partially dissolved in the salt and diffused onto the surfaces of MoB and Al_8_Mo_3_, and reacted to form MoAlB and Al-rich Al-Mo phases (Al_x_Mo), respectively. With increasing the temperature to >900 °C, the dissolution of B in the salt became more significant. So, the dissolved B would diffuse through the molten salt onto the surfaces of the intermediate Al–Mo phases formed earlier, and reacted with them to form additional MoAlB.
